# Effect of kidney donation on bone mineral metabolism

**DOI:** 10.1371/journal.pone.0235082

**Published:** 2020-07-07

**Authors:** Thomas F. Hiemstra, Jane C. Smith, Kenneth Lim, Dihua Xu, Shreya Kulkarni, J. Andrew Bradley, Kaido Paapstel, Inez Schoenmakers, John R. Bradley, Laurie Tomlinson, Carmel M. McEniery, Ian B. Wilkinson

**Affiliations:** 1 Cambridge Clinical Trials Unit, University of Cambridge, Cambridge, United Kingdom; 2 Department of Medicine, University of Cambridge, Cambridge, United Kingdom; 3 Division of Nephrology, Indiana University School of Medicine, Indianapolis, IN, United States of America; 4 Department of Medicine and Surgery, University of Cambridge, Cambridge, United Kingdom; 5 Endothelial Centre, University of Tartu, Tartu, Estonia; 6 School of Medicine, University of East Anglia, Norwich, United Kingdom; 7 London School of Hygiene and Tropical Medicine, London, United Kingdom; University of Pittsburgh School of Medicine, UNITED STATES

## Abstract

Kidney donation results in reductions in kidney function and lasting perturbations in phosphate homeostasis, which may lead to adverse cardiovascular sequelae. However, the acute effects of kidney donation on bone mineral parameters including regulators of calcium and phosphate metabolism are unknown. We conducted a prospective observational controlled study to determine the acute effects of kidney donation on mineral metabolism and skeletal health. Biochemical endpoints were determined before and after donation on days 1, 2 and 3, 6 weeks and 12 months in donors and at baseline, 6 weeks and 12 months in controls. Baseline characteristic of donors (n = 34) and controls (n = 34) were similar: age (53±10 vs 50±14 years, p = 0.33), BMI (26.3±2.89 vs 25.9±3.65, p = 0.59), systolic BP (128±13 vs 130±6 mmHg, p = 0.59), diastolic BP (80±9 vs 81±9 mmHg, p = 0.68) and baseline GFR (84.4±20.2 vs 83.6±25.2 ml/min/1.73m^2^, p = 0.89). eGFR reduced from 84.4±20.2 to 52.3±17.5 ml/min/1.73m^2^ (p<0.001) by day 1 with incomplete recovery by 12 months (67.7±22.6; p = 0.002). Phosphate increased by day 1 (1.1(0.9–1.2) to 1.3(1.1–1.4) mmol/L, p <0.001) but declined to 0.8(0.8–1.0) mmol/L (p<0.001) before normalizing by 6 weeks. Calcium declined on day 1 (p = 0.003) but recovered at 6 weeks or 12 months. PTH and FGF-23 remained unchanged, but α-Klotho reduced by day 1 (p = 0.001) and remained low at 6 weeks (p = 0.02) and 1 year (p = 0.04). In this study, we conclude that kidney donation results in acute disturbances in mineral metabolism characterised by a reduced phosphate and circulating α-Klotho concentration without acute changes in the phosphaturic hormones FGF23 and PTH.

## Introduction

Mineral and bone disorder (MBD) is a life-limiting complication in patients with Chronic Kidney Disease (CKD). The three component alterations that characterize MBD include disturbances in mineral metabolism, osteodystrophy and extraskeletal calcification. Abnormalities in mineral metabolism in CKD include the development hyperphosphataemia, elevations in the phosphatonins fibroblast growth factor (FGF)-23 and parathyroid hormone (PTH), reduced circulating active 1,25-dihydroxyvitamin D and hypo- or hypercalcaemia [1]. These pertubations become evident even with very mild decrements in glomerular filtration rate (GFR) [2]. Epidemiological data suggest that mineral disorders such as hyperparathyroidism and hyperphosphataemia are associated with increased morbidity and mortality, notably from cardiovascular disease (CVD) [3, 4]. In fact, the development of MBD and CVD are closely linked and understanding this complex relationship is of critical importance given that CVD remains the leading cause of death in patients with CKD [5–7].

Skeletal disorders in CKD are characterised by alterations of bone morphology and turnover that result in an increased fracture risk and may contribute to extraskeletal calcification [8]. Notably, CKD-MBD is associated with widespread vascular and soft-tissue calcification. Arterial calcification is closely linked to arterial stiffness [9] and contributes to the excess cardiovascular mortality observed in patients with renal impairment [10]. Both skeletal health and extraskeletal mineralization are intricately linked with calcium and phosphate homeostasis, which is predominanlty regulated by vitamin D, PTH, FGF23 and α-klotho. These modulators of bone mineral metabolism are responsive to changes in kidney function, and interact with each other and with calcium and phosphate concentrations through multiple endocrine feedback loops. Establishing the primary abnormality arising from a decrement in eGFR and driving abnormal mineral metabolism is important in order to guide the design of interventions to reduce the cardiovascular and skeletal sequelae of CKD-MBD [1].

Kidney donation represents an excellent model for studying the effects of a decrease in GFR on MBD, since it allows interrogation of the effect of an acute and isolated GFR reduction of 50% in the absence of significant systemic disease [11, 12] Further, given concerns over the cardiovascular sequelae of reductions in GFR [13, 14] (and hence the potential for adverse cardiovascular consequences of kidney donation) and the converse observation of a reduction in cardiovascular risk through kidney transplantation [15], there is an urgent need to identify the relationship between early changes in bone mineral metabolism markers and measures of cardiovascular and skeletal health.

We conducted a prospective, controlled study of living kidney donors and healthy controls to elucidate the effects of an acute decrement in GFR on biochemical markers of mineral metabolism, haemodynamic parameters and skeletal health.

## Methods

### Study design

**Fig 1:**
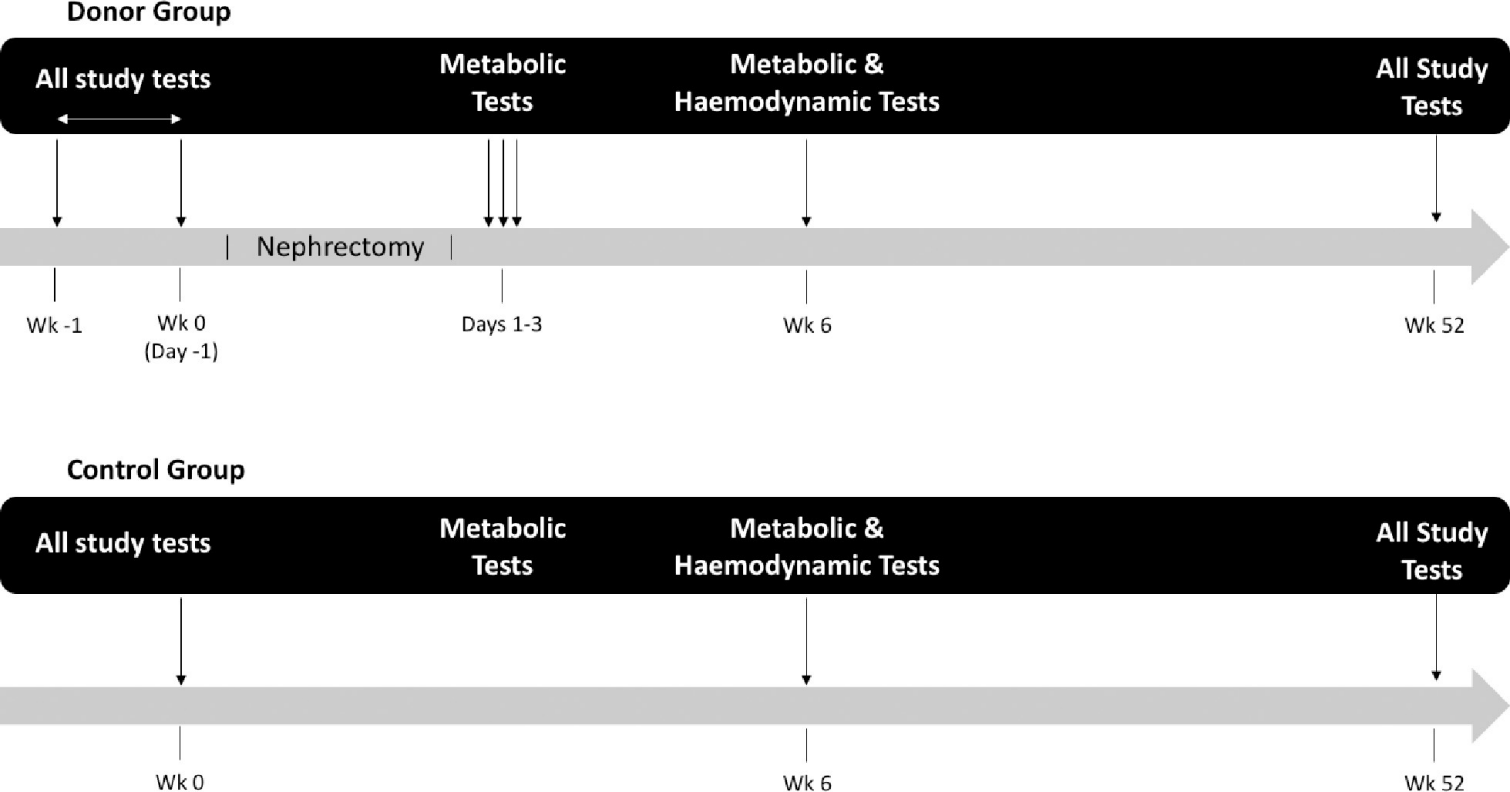
The KARMA study design.

The KARMA (Effect of Kidney donAtion on bone and mineRal MetAbolism) study was a single-center matched prospective cohort study that enrolled patients between May 2012 and March 2014 at Addenbrooke’s Hospital, Cambridge University Hospitals NHS Foundation Trust (CUH), United Kingdom. All kidney transplants were carried out in the UK National Health Service. The National Health Service is free at the point of care. Donors did not receive any payments. The KARMA study design is illustrated in Fig 1. All patients undergoing donor-nephrectomy were invited to participate. Biochemical parameters were determined on 6 occasions in the donor group before and acutely after donation on post-operative days 1–3, and 6 weeks and 12 months after kidney donation.

The control group was recruited from patients who volunteered to undergo donor-nephrectomy and were found to be medically suitable but did not proceed for non-medical reasons, such as a positive cross match or cadeveric transplant for the planned recipient. We sought to match control patients in terms of age and gender as far as possible. Patients with actual or planned pregnancy were excluded. Biochemical parameters were determined at baseline, 6 weeks and 12 months in the control group.

In both groups, cardiovascular parameters including ambulatory blood pressure and carotid-femoral pulse wave velocity were recorded. The donor group underwent bone mineral density (BMD) assessment by DEXA scanning at baseline and after 12 months. Bone density was determined by DEXA scanning on all kidney donor patients at baseline and 12 months. BMD assessment was not carried out in the control group.

### Laboratory investigations

Blood and urine samples in the immediate post transplant period (day 1 to day 3 after transplantation) were taken as early morning samples to ensure standardization. Biochemical analysis was conducted at Addenbrooke’s Hospital clinical laboratory and Cambridge Core Biochemical Assay Laboratory (CBAL). Creatinine, calcium, inorganic phosphate, albumin & alkaline phosphatase were analysed on the Siemens Advia 2400 autoanalyser. PTH [normal range 14–72 ng/l] was analysed using the ADVIA® Centaur XP Immunoassay system for intact PTH (1–84) The eGFR was computed using the four-variable Modification of Diet in Renal Disease equation. Serum FGF-23 levels were analyzed using an ELISA kit detecting the FGF-23 c-terminal (Immutopics; Cat #60–6100). This assay detects both the intact molecule and large carboxyl terminal fragments of human FGF-23. Serum α-Klotho levels were analyzed (Immuno-Biological Laboratories Co., Ltd., Cat #27998). 25-Hydroxyvitamin D and 1,25-Dihydroxyvitamin D were analysed by automated chemiluminescent immunoassay on the Diasorin Liaison XL autoanalyser.

### Ethical approval

The study protocol was approved by the NRES Committee East of England–Cambridge EAST (REC reference 10/H0304/74). All participants provided written informed consent. None of the transplant donors were from a vulnerable population and all donors provided written informed consent that was freely given.

### Kidney donation

Prospective kidney donors were seen on multiple occasions on separate dates in the outpatient clinic during the screening process and interviewed by a member of the transplant team and a transplant coordinator. Information about donation was provided in written and verbal form, with a full description of the procedure, the potential risks and complications and the nature and duration of the recovery period. Prospective donors were afforded as much time as required to come to a decision. All donors were living donors and were registered as organ donors. No organs were obtained from deceased donors. Kidney donation for transplantation followed a standard local protocol. Nephrectomy was performed by a laparoscopic technique. Donors received enoxaparin 40mg before surgery, flucloxacillin 1 gram and 100ml 20% mannitol intravenously intra-operatively, ibuprofen 200mg three times daily and paracetamol 1 gram 6 hourly as required in the post-operative period, and intravenous fluid as 0.9% Saline as required. Donors remained nil by mouth on the day of donation, but were allowed an oral diet from day 1 after donation. The majority of transplant donors were discharged from hospital on the second or third post-operative day.

### Statistical analysis

Data are presented as mean ± SD, median (IQR) according to their distribution. Counts are presented as frequencies (%). Between-group comparisons were performed using independent samples T tests or Mann Whitney U-tests as appropriate. Within-subject comparisons were made using repeated measures ANOVA, or chi-squared for categorical variables. Parameter estimate, standard error, and 95% confidence interval (CI) were calculated for each variable. P<0.05 was considered statistically significant. All analyses were conducted using STATA (version 14).

## Results

We enrolled 34 kidney donors and 34 controls between 2012 and 2016 at Addenbrooke’s Hospital, Cambridge.

### Clinical characteristics of the study population

Clinical and biochemical parameters between the donor and control populations were similar at baseline (Table 1). A similar proportion (20/34, 59%) of the donors and controls (16/34, 47%) were male. The mean age of donors was 53±10 years, and did not differ from controls (50±14 years, p = 0.33). There was no difference in BMI (26.3±2.89 vs 25.9±3.65 kg/m^2^, p = 0.59), systolic and diastolic clinic blood pressure (SBP 128±13 vs 130±13mmHg, p = 0.59; DBP 80±9 vs 81±9 mmHg, p = 0.68) between donors and controls. PWV was also not statistically different between the donor and control groups at baseline (7.2 (6.8–7.8) vs 6.6 (6.0–7.7) ms, p = 0.25). Baseline eGFR was similar between donors and controls (84.4±20.2 vs 83.6±25.2 ml/min/1.73m^2^, p = 0.89). Biochemical and hematologic parameters between groups, including phosphate (p = 0.97), PTH (p = 0.74) and hemoglobin (p = 0.19), were not statistically different. Donor patients had marginally higher albumin (41.0 (39–42) vs 39.0 (37–42) mg/l, p = 0.02) levels than control patients.

**Table 1:** Baseline characteristics.

Variables	Control	Donor	p-value
No. of subjects	34	34	-
Male, n (%)	20 (59)	16 (47)	0.33
Age, years	53±10	50±14	0.33
BMI	25.9±3.65	26.3±2.89	0.59
Systolic BP, mmHg	129.6±12.8	127.9±13.01	0.59
Diastolic BP, mmHg	81.21±9.46	80.3±9.15	0.68
Tobacco:			
- Smokers, n (%)	1 (2.9)	1 (2.9)	-
- Ex-smokers, n (%)	5 (14.7)	18 (52.9)	-
**Applanation tonometry:**			
- PWV, m/s	6.6[6.0, 7.7]	7.2[6.8, 7.8]	0.25
**Laboratory:**			
- eGFR, ml/min/1.73m^2^	83.6±25.2	84.4±20.2	0.89
- Creatinine, mg/dL	79.5±19.1	81.5±10.9	0.82
- Corrected calcium, mmol/L	2.19[2.13–2.22]	2.23[2.12–2.28]	0.001
- Phosphate, mmol/L	1.1[0.97, 1.15]	1.1[0.95,1.23]	0.97
- Albumin, mg/l	39.0[37,42]	41.0[39,42]	0.02
- PTH, pmol/l	4.4[3.71, 8.5]	4.8[3.39, 5.83]	0.74
- 25-vitamin D, ng/ml	19.6[17.0, 22.7]	21.5[16.2, 24.8]	0.77
- 1,25-vitamin D, ng/ml	44.8 [37.2, 49.2]	49.5[40.8, 56.7]	0.15
- α-Klotho, pg/ml	893.4[739.8, 1051.0]	931.1 [662.7, 1144.6]	0.69
- FGF-23, RU/ml	49.0[28.0, 81.0]	47.7[35.9, 55.8]	0.37
- Alkaline phosphatase, ALP	76.0 [64.0, 101.0]	97.5 [84.0, 111.0]	0.01
- hs-CRP, mg/ml	1.2 [0.50, 1.9]	1.8 [0.86, 2.9]	0.25
- Hemoglobin, g/dl	13.8[12.9,14.1]	13.9[13.2, 14.7]	0.19

Data are presented as mean ± SD, median [interquartile range] or frequencies (%). P-value was obtained by student t-test.

### Biochemical parameters

**Fig 2:**
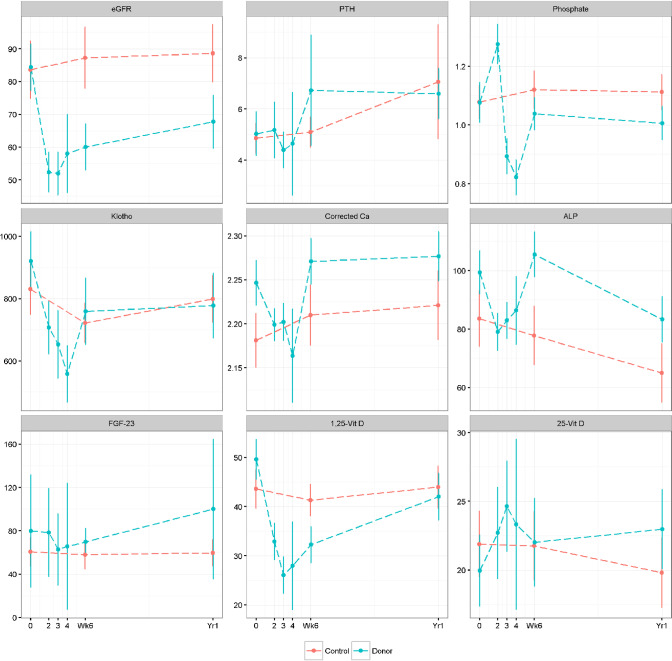
Changes in biochemical parameters over time. Legend: Changes in biochemical parameters from baseline at days 1, 2 and 3, 6 weeks and 12 months after donation in Donors (blue) and at 6 weeks and 12 months in Controls (red). Donation resulted in a marked and sustained reduction in eGFR (ml/min/1.73m^2^) (A), a striking increase followed immediately by a decrease in phosphate (C) despite a late and non significant change in intact PTH (pmol/l) (B) and no significant change in FGF-23 (RU/ml) (G). Klotho (pg/ml) reduced significantly immediately after donation and remained below baseline (D). Corrected calcium (mmol/L) reduced (E) in parallel to changes in 1,25-dihydroxyvitamin D (ng/ml) (H). Dashed lines illustrated mean or median, solid lines represent SD or IQR.

Kidney donation reduced eGFR markedly from 84.4±20.2 to 52.3±17.5 ml/min/1.73m^2^ (p<0.001) on post-operative day 1 (Table 2; Fig 2A). As expected, eGFR subsequently increased, but remained lower than baseline 6 weeks (60.0±20.0; p<0.001) and 12 months (67.7±22.6; p = 0.002) following kidney donation. eGFR was significantly lower in donors compared to controls at 6 weeks (p<0.001) and 12 months (p<0.001). Laboratory measurements early after kidney donation on post-operative days 1–3 are shown in Table 2 and mid-term changes at 6 weeks and 12 months are shown in Table 3. We will first describe acute mineral changes followed by mid-term changes.

**Table 2:** Laboratory measurements in the acute period after kidney donation.

Variables	Timepoint	Control	Donor	p-value^1^
- eGFR, ml/min/1.73m^2^	Baseline	83.6±25.2	84.4±20.2	-
	Day 1	-	52.3±17.5	<0.001
	Day 2	-	51.9±18.7	<0.001
	Day 3	-	58.0±22.4	<0.001
- Creatinine	Baseline	79.5±19.1	81.5±10.9	-
	Day 1	-	126.1±22.3	<0.001
	Day 2	-	128.6±25.7	<0.001
	Day 3	-	117.5±26.7	<0.001
- Corrected calcium, mmol/L	Baseline	2.25±0.1	2.3±0.07	-
	Day 1	-	2.2±0.05	0.003
	Day 2	-	2.2±0.06	0.008
	Day 3	-	2.2±0.09	0.002
- Phosphate, mmol/L	Baseline	1.1[1.0, 1.2]	1.1[0.9,1.2]	-
	Day 1	-	1.3[1.1, 1.4]	<0.001
	Day 2	-	0.9[0.8, 1.0]	<0.001
	Day 3	-	0.8[0.8, 0.9]	<0.001
- Alkaline phosphatase	Baseline	76.0[64.0, 101.0]	97.5[84.0, 111.0]	-
	Day 1	-	79.5[70.0, 88.0]	<0.001
	Day 2	-	80.0[73.0, 92.0]	<0.001
	Day 3	-	82.0[63.0, 104.0]	0.05
- PTH, pmol/l	Baseline	4.4[3.71, 8.5]	4.8[3.4, 5.8]	-
	Day 1	-	4.0[3.3, 6.5]	0.829
	Day 2	-	3.8[2.9, 5.4]	0.250
	Day 3	-	4.6[2.7, 5.5]	0.661
- 25-vitamin D, ng/ml	Baseline	19.6[17.0, 22.7]	21.5 [16.2, 24.8]	-
	Day 1	-	30.5[18.0, 53.0	0.19
	Day 2	-	36.5[16.0, 49.0]	0.03
	Day 3	-	48.0[39.0, 66.0]	0.23
- 1,25-vitamin D, ng/ml	Baseline	44.8 [37.2, 49.2]	49.5 [40.8, 56.7]	-
	Day 1	-	45.5[32.0, 63.0]	<0.001
	Day 2	-	30.0[14.0, 49.0]	<0.001
	Day 3	-	38.0[9.0, 57.0]	<0.001
- α-Klotho, pg/ml	Baseline	893.4[739.8, 1051.0]	931.1 [662.7, 1144.6]	-
	Day 1	-	677.7 [536.7, 833.9]	0.001
	Day 2	-	574.9 [470.0, 757.8]	<0.001
	Day 3	-	505.0[436.0, 638.4]	<0.001
- FGF-23, RU/ml	Baseline	49.0[28.0, 81.0]	47.7[35.9, 55.8]	-
	Day 1	-	51.1[39.2, 72.4]	0.97
	Day 2	-	36.1[31.1, 53.1]	0.57
	Day 3	-	32.9[28.5, 45.5]	0.75

Data are presented as means ± SD, median [interquartile range] or frequencies (%). P-value was obtained by repeated measures t-test. P-value^1^: Each P value was compared to baseline.

**Table 3:** Laboratory measurements mid-term after kidney donation.

Variables	Group	Baseline	6 weeks	12 months	p-value^1^
- eGFR, ml/min/1.73m^2^	Controls	83.6±25.2	87.2±25.9	88.6±24.3	0.568//0.416
	Donors	84.4±20.2	60.0±20.0	67.7±22.6	<0.001/0.002
	p-value^2^	0.893	<0.001	<0.001	
- Creatinine	Controls	79.5±19.1	76.5±16.0	73.5±18.9	0.328/0.008
	Donors	81.5±10.9	79.9±10.9	112.2±17.1	<0.001/<0.001
	p-value^2^	0.003	<0.001	0.006	
- Corr calcium, mmol/L	Controls	2.2±0.1	2.2±0.1	2.2±0.1	0.212/0.107
	Donors	2.2±0.1	2.3±0.1	2.3±0.1	0.182/0.112
	p-value^2^	0.001	0.005	0.02	
- Phosphate, mmol/L	Controls	1.1[0.97, 1.2]	1.1[1.0, 1.2]	1.1[1.1,1.2]	0.312/0.405
	Donors	1.11[0.95,1.2]	1.0[1.0, 1.13]	1.0[0.9, 1.1]	0.381/0.115
	p-value^2^	0.977	0.06	0.04	
- Alkaline phosphatase, IU/L	Controls	76.0[64.0, 101.0]	71.0[62.0, 81.0]	64.0[50.5, 76.0]	0.398/0.008
	Donors	97.5[84.0, 111.0]	106[89.0, 122.0]	85.0[66.5, 95.0]	0.247/0.003
	p-value^2^	0.010	<0.001	0.005	
- PTH, pmol/l	Controls	4.4[3.71, 8.5]	4.7[3.9, 6.3]	5.7[4.77, 7.1]	0.569/0.05
	Donors	4.77[3.39, 5.8]	5.3[4.1,7.2]	5.8[4.56, 8.7]	0.130/0.018
	p-value^2^	0.739	0.132	0.701	
- 25-vitamin D, ng/ml	Controls	23.4[20.5, 29.9]	19.5[17.3, 24.7]	23.0[16.55, 28.35]	0.134/0.287
	Donors	21.5[16.2, 24.8]	22.2[14.5, 29.8]	25.3[14.9, 28.2]	0.309/0.119
	p-value^2^	0.769	0.449	0.001	
- 1,25-vitamin D, ng/ml	Controls	41.9[35.3, 46.6]	41.4[35.7, 47.2]	42.4[32.8, 48.9]	0.627/0.985
	Donors	49.5[40.8, 56.7]	30.7[27.1, 36.7]	41.7[33.3, 50.0]	<0.001/0.016
	p-value^2^	0.148	<0.001	0.294	
- α-Klotho, pg/ml	Controls	893.4[739.8, 1051.0]	675.1[604.1, 814.7]	821.8[671.8, 1059.6]	-/0.345
	Donors	931.1 [662.7, 1144.6]	701.6[548.6, 874.0]	721.4[562.5, 956.5]	0.02/0.04
	p-value^2^	0.699	0.176	0.143	
- FGF-23, RU/ml	Controls	49.0[28.0, 81.0]	48.0[17.0, 72.0]	57.0[30.0, 83.0]	0.379/0.801
	Donors	47.7[35.9, 55.8]	60.2[50.9, 81.2]	64.0 [52.5, 83.4]	0.718/0.616
	p-value^2^	0.489	0.188	0.215	

Data are presented as means ± SD, median [interquartile range] or frequencies (%). P-value was obtained by student t-test. P-value^1^: 6 months vs baseline/12 months vs baseline in each respective group. P-value^2^: compares controls vs donors at each respective time-point.

**Fig 3:**
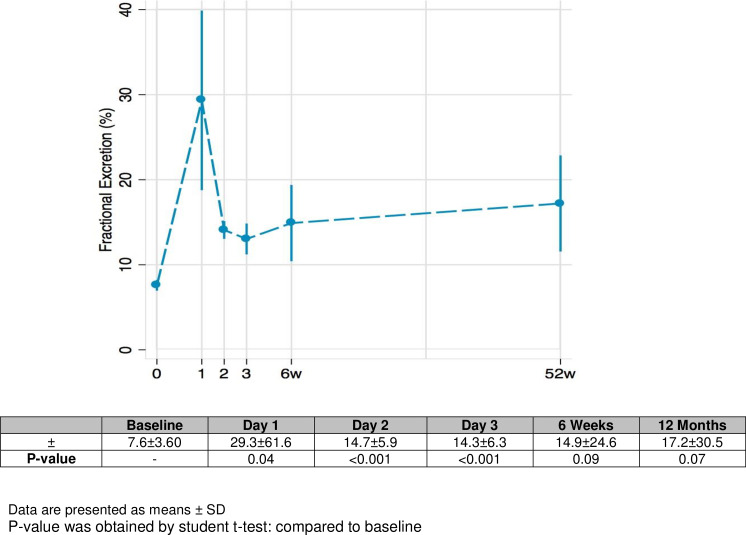
Fractional Excretion of Phosphate (FEP) in donors. Data are presented as means ± SD. P-value was obtained by student t-test: compared to baseline.

Acutely, albumin-corrected calcium concentration was reduced from 2.3±0.07 to 2.2 ± 0.05 mg/l (p = 0.003) by post-operative days 1 and remained low by post-operative day 3. Serum phosphate concentrations showed an increase from 1.1 (1.0–1.2) to 1.3 (1.1–1.4) mmol/L (p<0.001) by day 1 before declining sharply below baseline by day 2 (0.9 (0.8–1.0) p<0.001) and 3 (0.8 (0.8–0.9), p<0.001)(Fig 2C). The acute changes in phosphate not paralleled by any statistically significant changes in PTH levels from post-opertive days 1–3. However, these changes were associated with an increase in the fractional excretion (%) of phosphate from baseline (7.6±3.60) by day 1 (29.3±61.6, p = 0.04) post-operatively (Fig 3). Fractional excretion of phosphate reduced but remained elevated above baseline on day 2 (14.7±5.9, p<0.001) and day 3 (14.3±6.3, p<0.001) post-operatively. Alkaline phosphatase (ALP) reduced from 97.5 (84.0–111.0) to 79.5 (70.0–88.0) (p<0.001) by day 1 following donation (Fig 2F).

On mid-term follow-up, we found that albumin-corrected calcium concentrations t did not differ at 6 weeks or 12 months after donation (Fig 2E). Phosphate levels were not statistically different compared to baseline at 6 weeks (p = 0.381) or at 1 year (p = 0.115) after donation. However, PTH levels increased from 4.7 to 5.8 (4.6–8.7) pmol/L (p = 0.018) after 12 months, but was not statistically different after 6 weeks (Table 3, Fig 2B). There was no significant difference in fractionale excretion (%) of phosphate at 6 weeks (14.9±24.6, p = 0.09) and 12 months (17.2±30.5, p = 0.07) compared to baseline. ALP levels recovered to near baseline (p = 0.247; Table 3; Fig 2) after 6 weeks, but showed a further decline 12 months following kidney donation (p = 0.003).

### Changes in FGF-23, vitamin D and Klotho

Acutely, FGF-23 levels were unchanged from baseline (47.7 (35.9–55.8) RU/ml) to day 1 (51.1 (39.2–72.4) RU/ml, p = 0.97), day 2 (36.1 (31.1–53.1) RU/ml, p = 0.57) or day 3 (32.9 (28.5–45.5) RU/ml, p = 0.75) after kidney donation (Table 3; Fig 2G). Soluble α-klotho decreased markedly from 931 (662.7–1144.6) at baseline to 677.7 (536.7–833.9) (p = 0.001; Table 3; Fig 2) by day 1 following kidney donation (Fig 2D). Active vitamin D (1,25-dihydroxyvitamin D_3_) concentration decreased significantly by day 1 (p-<0.001; Table 3; Fig 2) and remained low by post-operative day 3. The concentration of 25-hydroxyvitamin D_3_ did not change after kidney donation (Fig 2I).

On mid-term follow-up, we found no change in FGF-23 levels at 6 weeks (48.0 (17.0–72.0) RU/ml, p = 0.379) or 12 months (57.0 (30.0–83.0), p = 0.616) after kidney donation. α-Klotho levels remained low after 6 weeks (701.6 (548.6–874.0), p = 0.02) and 1 year (721.4 (562.5–956.5), p = 0.04) following kidney donation. Active vitamin D levels remained at months (p<0.001) and at 12 months (p = 0.016) following kidney donation (Fig 2H). There was no change in inactive vitamin D at 6 months or 12 months follow-up.

### Bone density data

No difference in BMD (bone mineral density) was observed after 12 months compared to baseline at either the lumbar spine (L1 (p = 0.295), L2 (p = 0.717), L3 (p = 0.466) and L4 (p = 0.943)), femoral neck (p = 0.402), shaft (p = 0.510) or total hip region BMD (p = 0.436) (Table 4).

**Table 4:** DEXA parameters in donors.

	Baseline	12 months	p-value
**Lumbar spine BMD (g/cm**^**2**^**):**			
- L1	1.1±0.1	1.1±0.2	0.295
- L2	1.2±0.2	1.2±0.2	0.717
- L3	1.3±0.2	1.2±0.2	0.466
- L4	1.3±0.2	1.3±0.2	0.943
**Lumbar spine Z-score**			
- L1	-0.1±1.3	-0.2±1.4	0.807
- L2	0.0±1.3	0.1±1.4	0.829
- L3	0.7±1.5	0.6±1.5	0.917
- L4	0.7±1.7	0.8±1.9	0.698
**Femoral neck BMD (g/cm**^**2**^**)**	0.9±0.1	0.9±0.1	0.402
**Femoral neck Z-score**	0.0±0.9	0.0±0.8	0.992
		0.0±0.1^1^	
**Total BMD** (total hip region)	1.0±0.1	0.9±0.1	0.436
**Shaft BMD**	1.2±0.2	1.2±0.2	0.510

BMD: Bone Mineral Density

Data are presented as means ± SD

^1^Delta Z-score

P-value was obtained by student t-test: baseline vs 12 months

## Discussion

Kidney donation results in a predictable and marked decline in kidney function with incomplete recovery. We report the acute and medium-term changes in bone mineral metabolism after kidney donation compared to a well matched control population. Our data demonstrate that disturbances in mineral metabolism occur acutely following kidney donation and are detectable from the first post-operative day.

Unilateral nephrectomy resulted in a mean decline in eGFR of 38% after 24 hours, and eGFR remained 20% below baseline after 12 months. We found an acute elevation in serum phosphate (day 1), followed by an immediate and profound decline in phosphate to significantly lower than baseline by day 2. This was associated with an increase in fractional excretion of phosphate (_fe_PO4). It is therefore likely that the acute loss in GFR results in an initial reduction in phosphate excretion and hyperphosphataemia, which is (over) corrected by a phosphaturic response. Despite these observations, it is notable that we identified no detectable change in the phosphaturic factors PTH and FGF23 over the corresponding period. The driver for the increase in _fe_PO4 is not known.

**Fig 4:**
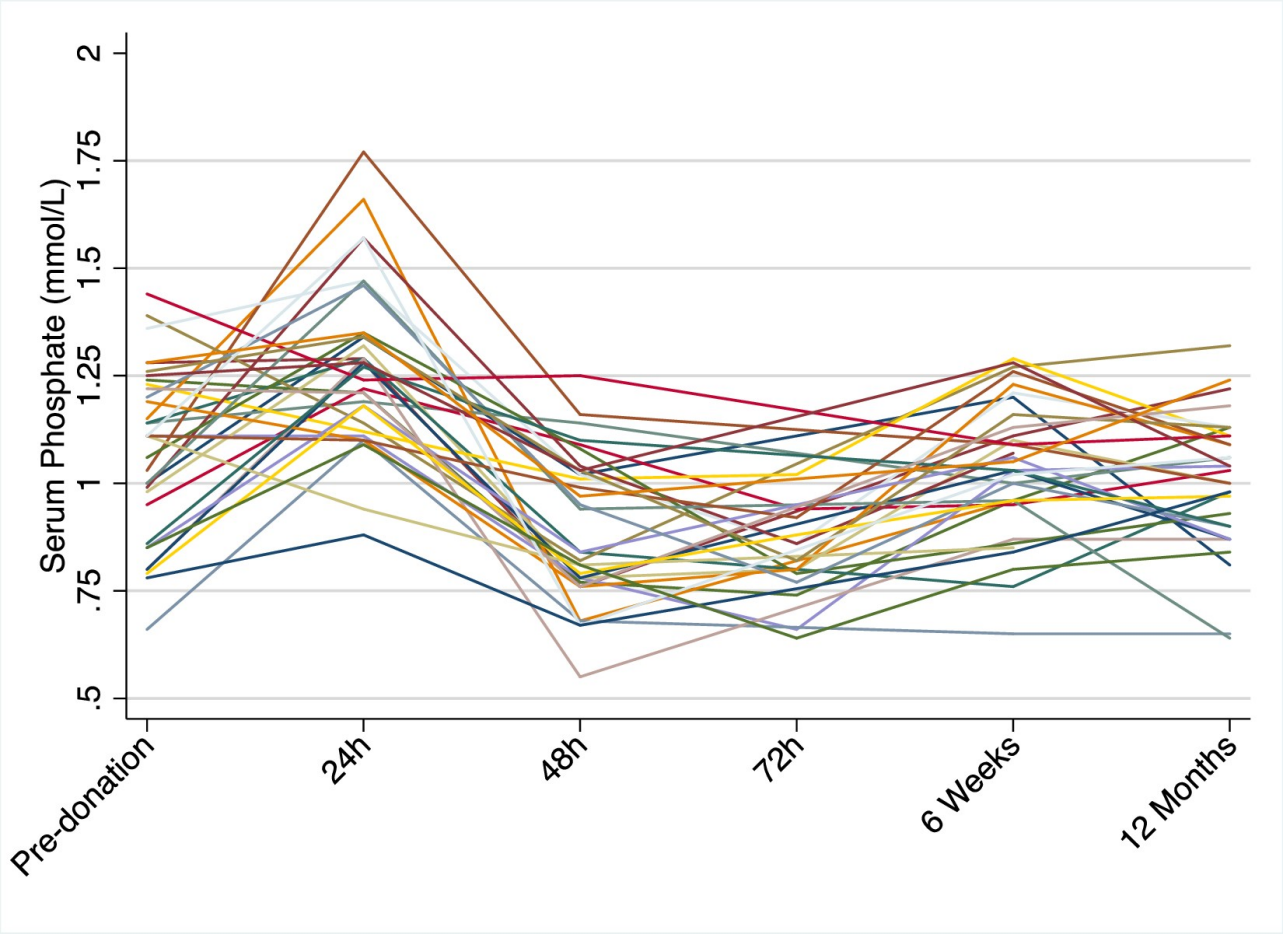
Individual patient phosphate concentrations.

In patients with CKD, hyperphosphatemia (and abnormal serum calcium levels) are usually not observed until the GFR declines to < 40mL/min in CKD patients [16]. However, progressive increases in PTH levels and decreasing serum vitamin D levels are seen in milder decrements in kidney function, typically when GFR is < 60ml/min. Our data showing an immediate increase in serum phosphate (Fig 2C, Fig 4), followed by a reduction in phosphate despite no acute change in PTH conflicts with a study by Ponte et al. which reported an increase in PTH 24 hours after donation without any increment in phosphate [17]. Further, while Ponte et al. reported no change in corrected calcium concentrations, we identified an immediate reduction in calcium. It is not clear why these two cohorts demonstrate differing patterns, but it is possible this may have arisen from differing peri-operative management regimes resulting from hydration, fasting and need for mineral repletion for example. The acute increment in phosphate and subsequent decline is unlikely to be attributable to the use of mannitol which is used to preserve living donor kidney preservation. Mannitol has a short half-life of approximately 20 minutes, and results in an acute increase in urinary phosphate excretion, mediated by PTH, and is therefore directionally opposite to our observations [18, 19]. Given our observation that FGF23 also did not change, the acute reduction in phosphate appears to be mediated through a PTH-FGF23 independent mechanism. This may be at least partly attributable to acute reductions in 1,25-dihydroxyvitamin D, resulting in reduced intestinal and renal tubular phosphate absorption.

We observed numerical but non-significant increases in PTH after 6 weeks, but PTH was no different between donors and controls after 12 months. In contrast, Kasiske et al. observed significant elevations in PTH after 6 months (for which we do not have a corresponding time point) and 36 months [20]. The absence of significant increases in PTH in our study may be explained by sample size (a larger sample size may have resulted in significance after 6 weeks), but is difficult to interpret given that time points between these studies do not overlap [17, 21].

In contrast to phosphate, we did not record any similarly biphasic changes in any other measured parameters over the first 72 hours. We also did not identify any change in bone density within the follow-up period (Table 4). However, we noted progressive decline in circulating 1,25-dihydroxyvitamin D, corrected calcium, alkaline phosphatase and circulating klotho. The absence of a change in bone density should be interpreted with caution since bone loss is slow in this age group and our study was relatively short in duration and not powered to detect differences in bone density.

We found that Klotho levels fell acutely by day 1 post-operatively and remained lower than baseline by 12 months following kidney donation. Klotho is produced predominantly by the kidney, which is thought to be the major source of circulating Klotho [22, 23] Circulating Klotho is phosphaturic, through direct inhibition of the apical membrane sodium-coupled phosphate transporter, NaPi-2a [24]. The results of our study challenge existing paradigms in mineral homeostasis; our data provide evidence that hypophosphatemia can develop in the absence of serum changes in the phosphaturic hormones, PTH and FGF-23 and despite declining serum Klotho levels. We postulate that the absence of rising FGF-23 levels in the acute phase following a decline in GFR may help counteract the development of further hypophosphatemia.

The strengths of the present study is the inclusion of a well-matched control group to comprehensively assess the acute changes in markers of bone and mineral metabolism. Participants were representative of the typical living kidney donor in the United Kingdom, and no participants were lost to follow-up. The results of the present study should be cautiously interpreted in light of its limitations. We recognize several limitations that include a relatively modest sample size and, to date, short follow-up. In the present study, given that the FGF23 assay used detects both the intact and c-terminal FGF23 fragments, we are unable to infer changes in the levels of the intact (active) or cleaved (inactive) FGF23 fragments. Additionally, due to current limitations in the availability of robust assays for assessing circulating Klotho levels, assessment of Klotho levels must be cautiously interpreted. To give an example of the magnitude of discrepancy between various assays, in one published study comparing a time-resolved fluorescence immunoassay (TRF; Cusabio, China) with an ELISA based assay (IBL, Japan), no correlation was found between the assays [25].

In summary, we identify biphasic acute changes in phosphate, and acute reductions in circulating klotho, 1,25-dihydroxyvitamin D and calcium. Larger studies with longer follow-up are required to establish the association between these changes and long term outcomes. Further physiological studies are required to establish the basis for the acute changes observed here.

## Supporting information

S1 File Inter- and intra-assay variations. (DOCX)

S1 Data (DTA)

S2 Data (DTA)

S3 Data (DTA)
