# Supplementary material for: Effect of kidney donation on bone mineral metabolism
Source: PLoS One. 2020 Jul 7;15(7):e0235082. doi: 10.1371/journal.pone.0235082 (PMC7340316; doi:10.1371/journal.pone.0235082)
Supplement: S1 File — (DOCX) [file pone.0235082.s001.docx]

**SUPPLEMENTAL MATERIALS**

Supplemental Table 1: Inter- and intra-assay variations.

1. **1,25 Hydroxy Vitamin D (Diasorin Liaison XL assay)**

There are no intra-assay CV’s supplied by Diasorin

Inter-assay Coefficients of Variation:

| 1-25OH VitD | Inter |
| --- | --- |
| pg/ml | %CV |
| 30.9 | 3.8 |
| 76 | 4.1 |
| 122.9 | 3.6 |

1. **1-84 PTH (Diasorin Liaison XL assay)**

There are no intra-assay CV’s supplied by Diasorin

Inter-assay Coefficients of Variation:

| PTH | Inter |
| --- | --- |
| pg/ml | %CV |
| 28.5 | 7.2 |
| 288.5 | 5.6 |
| 876.6 | 4.4 |

1. **Alpha KLOTHO (IBL ELISA kit)**

| alpha KLOTHO | Intra |
| --- | --- |
| pg/ml | %CV |
| 186.6 | 3.5 |
| 757.3 | 2.7 |
| 2969 | 3.1 |
|  |  |
| alpha KLOTHO | Inter |
| pg/ml | %CV |
| 165.5 | 11.4 |
| 706.3 | 6.5 |
| 2903 | 2.9 |

1. **FGF-23 (Immunotopics ELISA kit)**

| FGF-23 | Intra |
| --- | --- |
| U/ml | %CV |
| 33.7 | 2.4 |
| 302 | 1.4 |
|  |  |
|  |  |
| FGF-23 | Inter |
| U/ml | %CV |
| 33.6 | 4.7 |
| 293 | 2.4 |
|  |  |
